# Thoracolumbar transitional vertebrae: Quantitative differentiation and associated numeric variation in the vertebral column using skeletal remains

**DOI:** 10.1111/joa.13865

**Published:** 2023-04-06

**Authors:** Anneli M. Poolman, Quenton Wessels, Albert Van Schoor, Natalie Keough

**Affiliations:** ^1^ Division of Anatomy, School of Medicine University of Namibia Windhoek Namibia; ^2^ Department of Anatomy, School of Medicine, Faculty of Health Sciences University of Pretoria Pretoria South Africa; ^3^ Department of Anatomy and Cellular Biology, College of Medicine and Health Sciences Khalifa University Abu Dhabi United Arab Emirates

**Keywords:** anatomical variation, congenital, malformations, superior articular process

## Abstract

Transitional vertebrae at the thoracolumbar region are called thoracolumbar transitional vertebrae (TLTV) and retain physical features from the thoracic and lumbar regions. Since TLTV were first classified 40 years ago, there has been much discrepancy regarding its features, identification and clinical relevance. Vertebral body levels are used in the medical field as a frame of reference to locate specific organs, vessels, nerves or landmarks. Any numeric variation or deviation in the vertebral column may lead to clinical errors. Previous findings have suggested a high association between numeric variation and the presence of TLTV. Therefore, the aim of this study was to identify the types of TLTV observed and to identify any possible associated numeric variation in the vertebral column. This study also aimed to validate the established technique to quantitatively differentiate TLTV from T12 and L1 at the thoracolumbar junction using skeletal remains from a South African population group. Skeletal remains (*n* = 187) remains from the Pretoria bone collection were assessed. Measurements were taken of the angle of the superior zygapophyseal processes of the last thoracic vertebra (T12), the first lumbar (L1), and identified TLTV. The results indicate a TLTV prevalence of 35% (*n* = 66/187). The results show that each vertebral type (T12, L1, TLTV) fall into independent confidence intervals: T12 is 188° ± 9.22 (CI: 187° < μ < 189.6°), 110° ± 7.52 (CI: 109.2° < μ < 111.3°) in L1, and 135° ± 24.51 (CI: 130.4° < μ < 139.1°) in the TLTV. This study observed that 70% of cases with TLTV was associated with numeric variation in the spine, both homeotic and meristic and that TLTV has a 35% prevalence. The results clearly show that quantitative morphometric analysis can effectively differentiate TLTV from other vertebral types at the thoracolumbar junction in skeletal remains.

## Graphical abstract

Posterior view of thoracolumbar transitional vertebraes (TLTVs) demonstrating (a) asymmetry of superior facets, (b) aplasia or hypoplasia of transverse processes and (c) mammillary processes in the thoracic region. The images clearly demonstrate that TLTV resemble both the thoracic and lumbar regions. TLTV can be identified in the thoracic or lumbar region by using quantitative morphometrics.

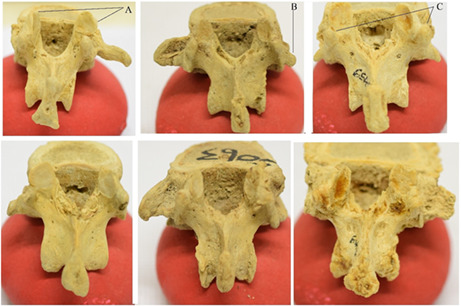

## 1 INTRODUCTION

Transitional vertebrae are anomalous vertebrae that result from overlapping somites, and are located at a regional junction in the vertebral column and retain features from the respective, adjacent vertebral regions (Bakker et al., 2017; Oostra et al., 2005). Originally it was stated, over 40 years ago, that thoracolumbar transitional vertebrae (TLTV) can be identified by the presence of at least one corresponding hypoplastic rib on the last rib bearing segment (Carrino et al., 2011; Doo et al., 2020; Park et al., 2016; Wigh, 1980). However, this classification often disregards TLTV located in the lumbar region and also may not be effective during osteological evaluation–predominantly used in anatomical research—as post mortem loss and damage of bones might not allow hypoplastic ribs to recovered and thus be observed (Buikstra & Ubelaker, 1994).

TLTV can be overlooked during clinical evaluation or medical procedures and have been suggested to be associated with numeric variation in the vertebral column. As the vertebral body levels is the key frame of reference used to identify anatomical landmarks, structures, vessels and organs it is critical to consider any numeric deviation in individuals (Jagannathan et al., 2017). Deviations in the vertebral segments will render the references null and void, as the vertebral numbering will be altered. Types of numeric variation that have been described in literature include homeotic and meristic transformations. Meristic transformations are variations in the number of vertebral segments in a specific region, without altering the number of segments in the adjacent regions. Alternatively, the change of one series identity at the expense of another is known as a homeotic transformation (Asher et al., 2011).

Recent findings have demonstrated that TLTV can clearly be distinguished by overlapping thoracic and lumbar features. According to these authors, TLTV can be classified into a four‐type system and can be identified qualitatively by overlapping features, more specifically the superior articular facets. The authors reported that the superior facets of TLTV are asymmetric, resembling both thoracic and lumbar regions. Alternatively, the superior facets of a TLTV can be orientated at an angle that lies in between that of the thoracic and lumbar vertebrae. Other features of TLTV that were observed in that study are aplasia/hypoplasia of the transverse processes and mammillary processes in the thoracic region or placed on the pedicle rather that the superior articular process in the lumbar region (Du Plessis et al., 2018).

As subjective observations leave much room for error, TLTV were recently quantitatively differentiated in a sample representative of the Namibian population using CT scans and this study found that although hypoplastic ribs were observed sometimes, it was not an identifying feature of TLTV (Du Plessis et al., 2021).

The aim of this study was to validate the confidence intervals used to differentiate vertebral types at the thoracolumbar junction in the South African population and also to evaluate whether the technique can be applied to skeletal remains. This is important especially during the evaluation of human skeletal remains in a forensic and archaeological setting. For any anthropological based research, clinical procedures or consultation in a forensic setting to be done accurately, correct inventory of all bones and sequencing of vertebrae and ribs are critical. As the TLTV has been reported to be associated with numeric variation in the vertebral column, this study also aimed to identify the types of TLTV and any numeric variation in the vertebral column. These changes should be kept in mind during any evaluation as this deviated from the typically expected vertebral segment distribution among the regions.

It is well established that typical vertebrae differ significantly in morphology and size between the respective vertebral regions (Chang et al., 2007; Doherty & Walker, 2014; McDonald, 2007; Rawls & Fisher, 2010; Watts et al., 2012; Wilson et al., 2014). In the thoracic region, the superior and inferior articular processes are typically oriented in the coronal plane and in the lumbar region are orientated in the sagittal plane (Forseen et al., 2015). By measuring the angles of the superior articular facets, one can quantitatively differentiate between vertebrae at the thoracolumbar junction.

## 2 MATERIALS AND METHODS

### 2.1 Materials

Skeletal remains from the Pretoria bone collection housed at the University of Pretoria were assessed. The sample (*n* = 187) included a random distribution of adults (32 Females; 155 Males) between the ages of 18 and 87 years old (mean = 58 ± 15.4); representative of the modern Gauteng population in South Africa. This sample size is considered sufficient for a 95% confidence interval and a margin of error of 7% for the Pretoria population. Ethical clearance from the Research Ethics Committees of the University of Pretoria (678/2018), which conforms to the principles within the Declaration of Helsinki (1964) and the National Health Act (2003) was obtained. Inclusion criteria included vertebral columns that were not discernibly damaged and that had complete regional junctions. Remains were not excluded based on sex and ethnic background. Any skeletal remains that demonstrated severe post‐mortem damage and/or post‐mortem loss of relevant bones or signs of trauma were excluded from the study.

Additional tools used to record the quantitative data in this study included an electro‐optical system digital camera, a tripod, Microsoft Excel®, and Digitize® imaging analysing software.

### 2.2 Methods

**FIGURE 1:**
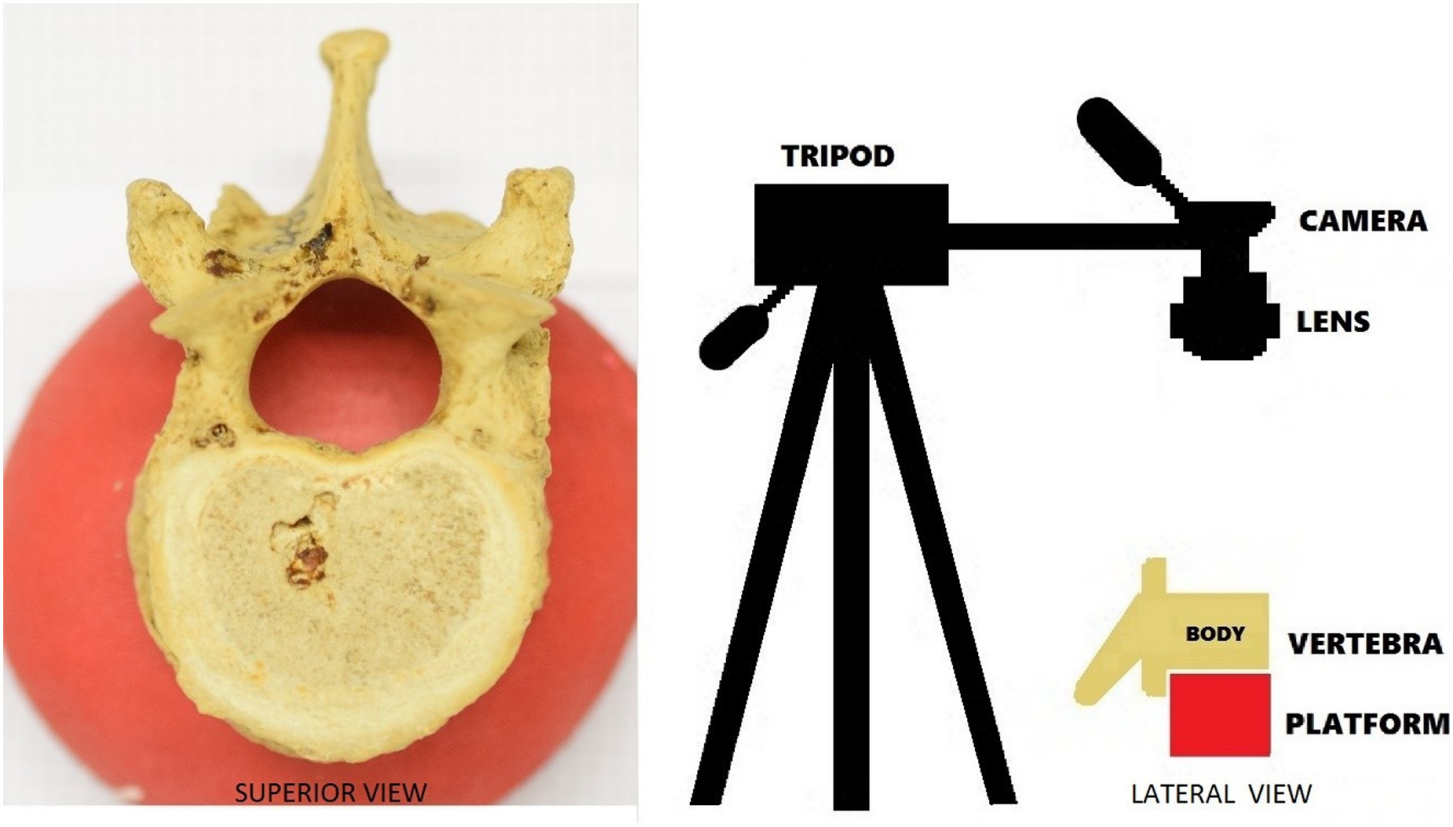
Example of high resolution image capturing method.

The measurements that were taken in this study were based on the most prominent feature of TLTV, specifically the variations in superior articular facet orientation. TLTV were identified by their overlapping qualitative features such as superior facet placement that is not typical for the region it is located in (posteriorly for thoracic vertebrae and medially for lumbar vertebrae). Attention was also paid to one mammillary bodies that were not located on the ends of the superior articular processes of lumbar vertebrae and hypoplasia/aplasia of the transverse processes. Additional measurements of regular vertebrae (T12 and L1) were taken to construct a baseline. To start, standardized photos of each vertebra were taken from a superior view using a high‐resolution camera and a tripod (Figure 1). Special attention was paid to make sure that the lens of the camera is parallel to the superior surface of the vertebral body. This was aided by placing the vertebral body on a platform, as the spinous process and articular processes prevented the vertebra from staying in position on a flat surface.

**FIGURE 2:**
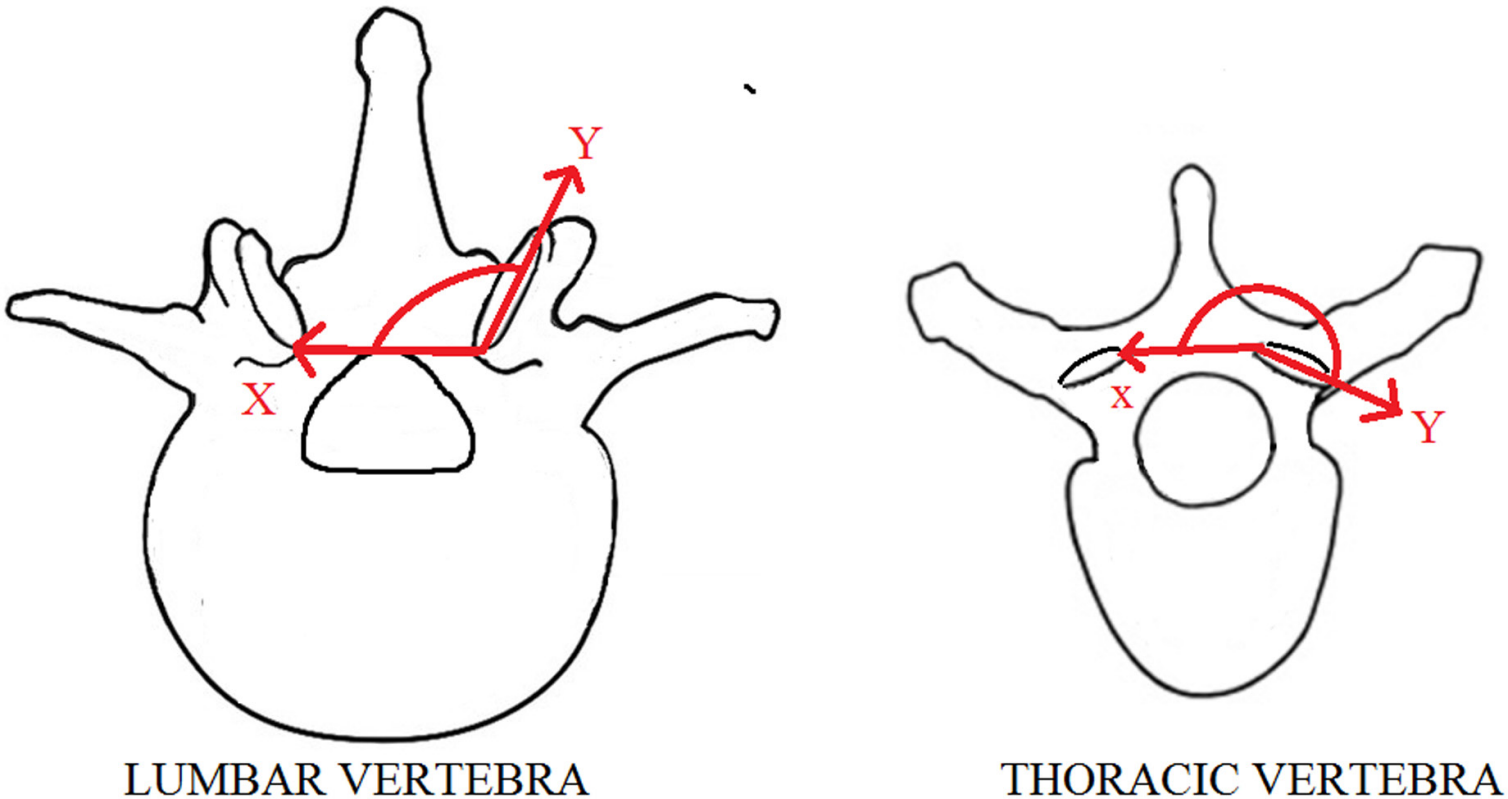
Superior view of a vertebra illustrating the measured angle of the superior articular facet with line “*X*” that runs between the two inferior points of the facet and line “*Y*” that runs directly adjacent to the articular surface of the facet Composite bell curve illustrating the independent distributions of vertebrae at the thoracolumbar junction Posterior view of thoracolumbar transitional vertebraes (TLTVs) demonstrating (a) asymmetry of superior facets, (b) aplasia or hypoplasia of transverse processes and (c) mammillary processes in the thoracic region. Superior views of TLTVs demonstrating superior facet asymmetry An illustrated bell curve depicting the classification of a vertebra based on coordinate location relative to the population distribution.

The digital images were subsequently imported into the imaging analysing software (Digitize®). With the appropriate software, measurements of the superior facet angle were recorded and the data exported to Microsoft Excel® for statistical analyses. The angles of both the left and right superior articular facets were measured. The angle measured is formed by a line that runs between the two most medial points of each superior facet (line X) and a line that runs directly adjacent to the articular surface of the facet that is being measured (line Y) (Figure 2). If the angle is larger than 180° it may require manual calculation to determine the angle size of the superior facets from most of the measurements taken in the thoracic region, depending on the image software used. Therefore, the calculation (angle = 360°‐ measurement; 270° > angle > 180°) was used.

Inter observer correlation analyses were used to assess the repeatability of the measuring system (*n* = 11). The results were analysed using correlation analyses and cross evaluated with the measurements taken by the primary researcher.

This study also recorded the different types of TLTV identified as well as any possible numeric variation in the vertebral column.

### 2.3 Statistical analyses

The following standard statistical comparisons were made: mean or average of each measurement; the standard deviation; 95% confidence interval, including upper and lower ranges; analysis of variance to determine the significance of variation (*p*‐value of <0.05 will be regarded as statistically significant). The Pearsonian coefficient (SK) values were calculated to determine whether there is a regular distribution of data.

## 3 RESULTS

### 3.1 Quantitative differentiation

**FIGURE 3:**
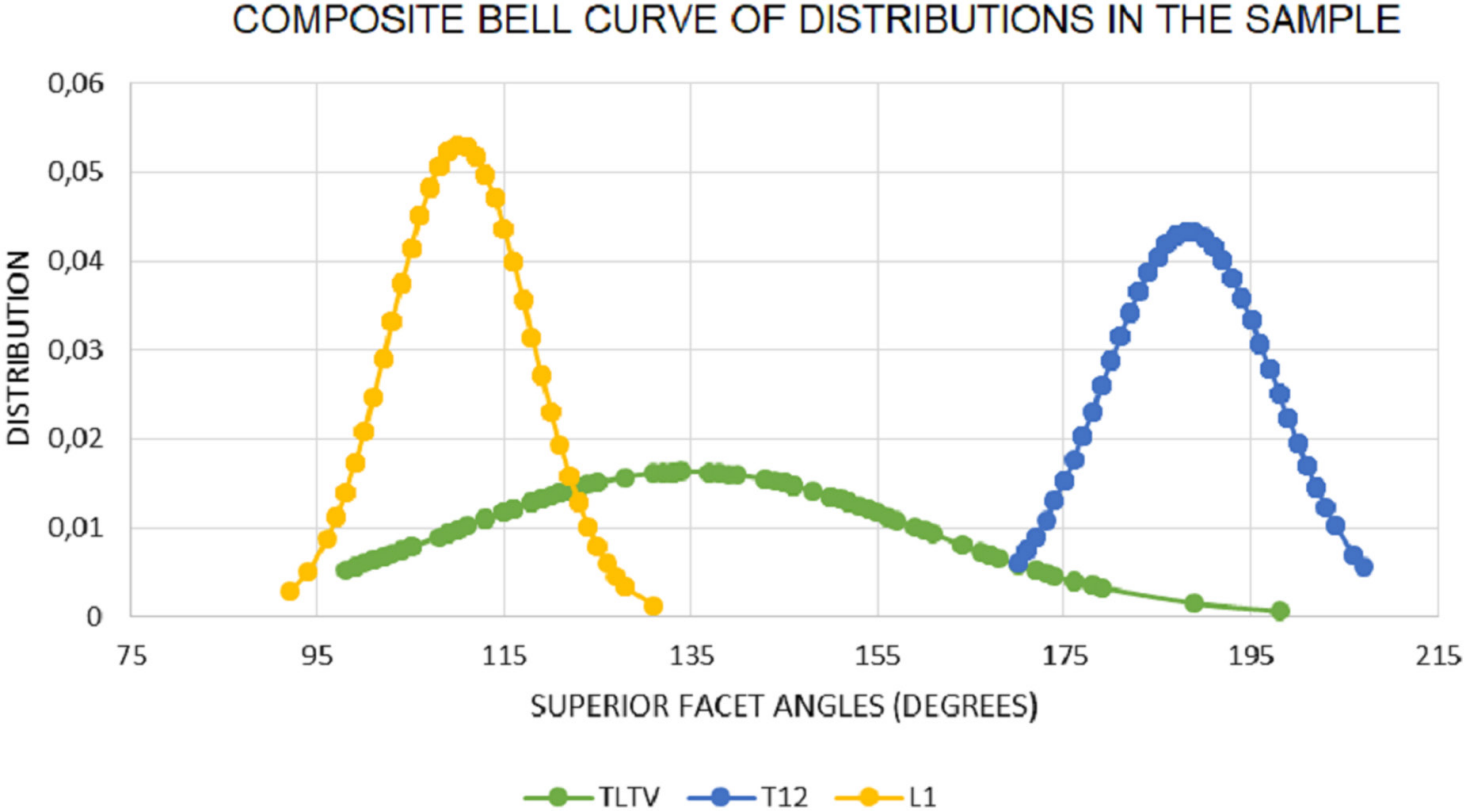
Composite bell curve illustrating the independent distributions of vertebrae at the thoracolumbar junction.

The results show that all the groups in the sample (*n* = 187) have distributions that fall within acceptable regular parameters T12 (SK = +0.11), L1 (SK = +0.29) and TLTV (SK = +0.40). When the distributions were plotted as bell‐curves (Figure 3), it is very clear that each vertebral type falls within completely separate distributions. The results show that in the sample, TLTV had aprevalence of 35%.

There was no significant difference between the right and left superior facet angles in regular T12 (*p* = 0.66) and regular L1 (*p* = 0.94), therefore implying a significant degree of symmetry in regular vertebral segments. The data for regular vertebrae were therefore pooled for descriptive summary statistics. This is consistent with the results from sample A.

Unlike regular vertebral segments, a significant difference (*p* < 0.05) was observed between the superior facets in TLTV when the facet resembling the thoracic region was compared to the facet resembling the lumbar region (Table 1). This, as previously mentioned, demonstrates the asymmetry of TLTV that is typically not seen in T12 and L1.

**TABLE 1:** Analysis of variance (ANOVA) between the right and left superior facet angles in thoracolumbar transitional vertebrae (TLTV).

ANOVA: TLTV						
Groups	Count	Sum	Average	Variance		
“thoracic like” facet	62	9088	146.58	537.23		
“lumbar like” facet	62	7628	123.03	390.98		
ANOVA						
Source of variation	SS	df	MS	*F*	*p‐*Value	*F* crit
Between groups	17,190.32	1	17,190.32	37.04	1.38 × 10^−8^	3.92
Within groups	56,621.03	122	464.10			
Total	73,811.35	123				

The results show that the average superior facet angle of T12 is 188° ± 9.22 (CI: 187° < μ < 189.6°), 110° ± 7.52 (CI: 109.2° < μ < 111.3°) in L1, and 135° ± 24.51 (CI: 130.4° < μ < 139.1°) in the TLTV. These categories clearly places the average angle size each vertebral type into a separate mean confidence interval (Table 2), as is similarly seen in sample A.

**TABLE 2:** Summary statistics of the sample.

Descriptive summary statistics	T12 group B1	L1 group B2	TLTV group B3
Mean	188.35	110.295	134.83
Standard deviation	9.22	7.52	24.51
Minimum	170	92	97.59
Maximum	207.42	131.19	197.97
Count	192	192	124
Pearson's coefficient (SK)	0.11	0.29	0.40
Confidence interval lower bound	187.04	109.23	130.48
Confidence interval upper bound	189.66	111.36	139.19
Confidence level (95.0%)	1.31	1.0698	4.36

Inter‐observer error analyses were used to assess the repeatability of the proposed quantitative model. The results indicated high correlation coefficients for measurements taken at T12 (0.933 < *r*_T12_ < 0.947) and also for measurements taken at L1 (0.919 < *r*_L1_ < 0.945). Showing that the proposed model can be repeated by other observers and that the measurements, when taken correctly, correlate highly with one another.

### 3.2 Classification of TLTV

This study identified a total of 66 TLTV within the sample of 187, relating to a prevalence of 35% in the Pretoria population of South Africa.

This study observed all classified TLTV types, with type L_1a_TLTV (*n* = 24) being the most frequent. This TLTV type is characterised by a TLTV located in the lumbar region without altering the number of lumbar vertebrae present. In many cases, no segment variation (*n* = 20) was observed in other vertebral regions (C7; T12; L5: S5) in L_1a_TLTV. Rare cases demonstrated completely sacralisation (C7:T12; L4; S6) (*n* = 2) and additional sacral vertebrae (C7; T12; L5; S6)(*n* = 2).

T_12_TLTV was the second most frequent (*n* = 20) TLTV type observed. This type of TLTV is identified when the TLTV is located on the 12th thoracic vertebrae without altering the number of thoracic vertebrae present. Some of the cases with T_12_TLTV had no segment variation (C7, T12, L5, S5) in the vertebral column (*n* = 15), complete sacralisation of L5 (C7; T12; L4; S6) (*n* = 4) and congenitally only four lumbar segments present (C7; T12; L4; S5) (*n* = 1).

T_13_TLTV was also recorded in the sample (*n* = 14). It was observed the (*n* = 10) of the T_13_TLTV types were additional vertebral segments at the thoracolumbar junction (C7; T13; L5; S5) and the remainder resulted from the cranial shift of L1 (C7; T13, L4; S5) (*n* = 4).

Lastly, L_1b_TLTV types were observed in 8 of the cases in the sample. It is characterised by a TLTV present on L1 when six lumbar vertebrae are present. It was observed that 6 of the L_1b_TLTV types resulted from an additional vertebral segment at the thoracolumbar junction (C7; T12; L6; S5) and that the remaining two (*n* = 2) cases resulted from complete lumbarization of the S1 segment (C7; T12; L6; S4).

## 4 DISCUSSION

The results from this study demonstrate a 24.5% prevalence of numeric variation in the vertebral column and a 35% prevalence of TLTV. This relatively high prevalence should raise awareness that variation in the vertebral segment distribution directly affects the numbering and frame of reference to identify critical anatomical structures. It is often assumed that the last vertebral segment with rib articulation is T12, however this study clearly demonstrated that this is not always the case. It is important to consider all vertebral segments in at least the thoracic, lumbar and sacral regions during clinical treatment or evaluation. The findings from this study also relate to the fields of nuclear science (radiotherapy), anatomical research and forensic anthropology. Any numeric variation influences the inventory and can be seen as an identifying feature of an individual. This may include cranial or caudal border shifts, additional vertebral segments or a diminished number of vertebral segments.

Based on the results, this quantitative model has shown to be effective in differentiating TLTV from other vertebral types at the thoracolumbar junction in skeletal remains. The TLTV may be located in either the thoracic or lumbar regions. The bell curves constructed in this study can be used to differentiate vertebrae at the thoracolumbar junction using the following steps.

**FIGURE 4:**
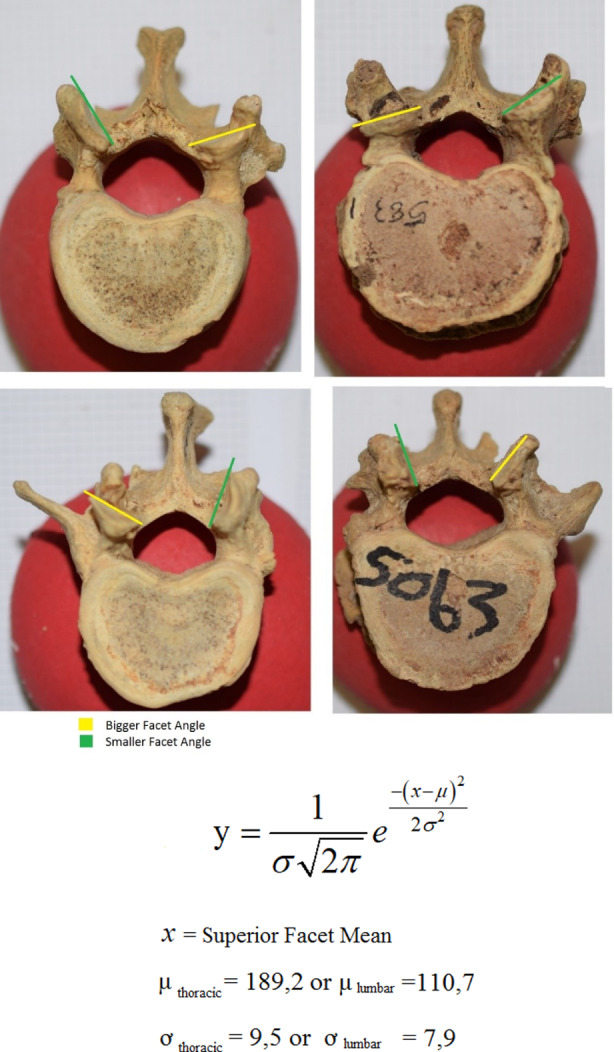
Superior views of thoracolumbar transitional vertebraes demonstrating superior facet asymmetry.

**FIGURE 5:**
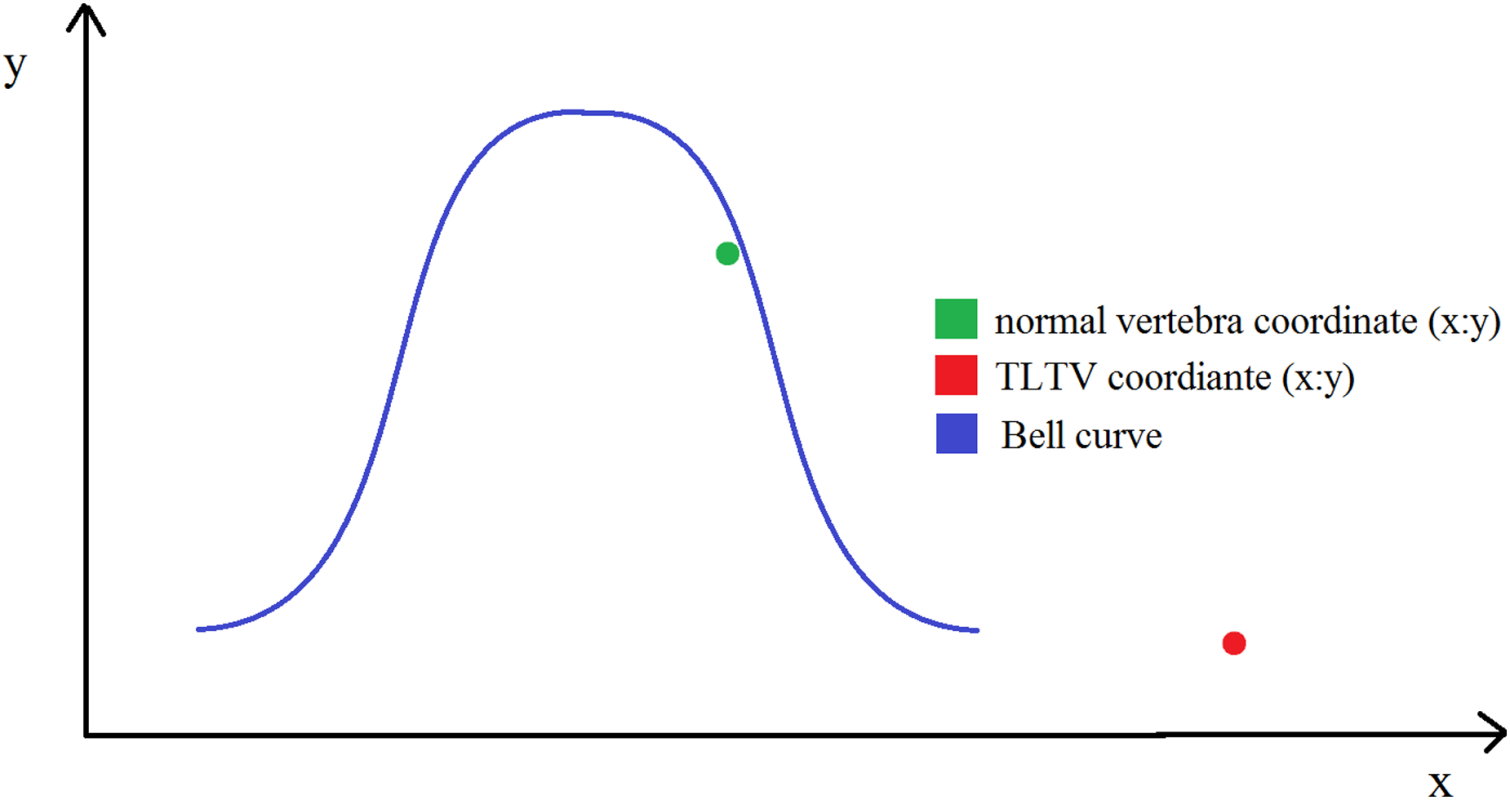
An illustrated bell curve depicting the classification of a vertebra based on coordinate location relative to the population distribution.

**STEP 1**: Take both measurements for the left and right superior facets and calculate the mean (*x*).
**STEP 2**: Calculate (*y*) coordinate using (*x*). Substitute the mean superior facet measurement (*x*) into the function below (Figure 4), to calculate the relative distribution that will be the (*y*) coordinate of the point (*x*:*y*).
**STEP 3**: Identify in which region the vertebra is situated.
If the vertebra has costal articulation it falls into the thoracic region and if the vertebra does not have costal articulation, it will fall into the lumbar region. The question to be answered now, is whether the vertebra is normal or a transitional vertebra, keeping in mind that transitional vertebrae can be located in either the thoracic or lumbar regions. If the vertebra in question falls into the thoracic region it should be compared to the thoracic T12 baseline, therefore, the T12 bell curve is used. Alternatively, if the vertebra is located in the lumbar spine, the bell curve for normal L1 should be used as the baseline. The baselines were constructed by pooling the measurements of normal T12 and L1 segments in the Western Cape and Gauteng samples.
**STEP 4**: Plot the coordinate onto the correct bell curve.
The vertebra in question can be quantitatively differentiated by plotting (*x*:*y*) coordinates onto the respective bell curves. If the coordinate point falls within the respective bell curve, it is considered a “normal” vertebra (T12 or L1). However, if the coordinate falls outside of the bell curve, the vertebra is a TLTV (Figure 5).

Confidence interval, in this case, specifically refers to the mean, which is a measure of central location. The results clearly infer that each of the means of each vertebral type are categorised into independent confidence intervals. Therefore, the mean superior facet measurements of TLTV fall outside the confidence interval of T12 and L1 segments; creating a unique category of its own. Therefore, the superior facet measurement of TLTV falls outside the confidence interval of T12 and L1 segments, creating a unique category of its own. For the CI method to be used, the average of several vertebral segments must be calculated after measuring the right and left superior facet angles. This value, referred to as (*x*), can be compared to the T12, L1 and TLTV CI intervals of the pooled population parameters. The confidence interval in which the value (*x*) falls, determines the type of vertebra it is.

In order to consider all data, the range must also be considered as it demarcates the superior facet interval in which point measurements taken of vertebrae in the thoracic and lumbar regions will lie. The results also demonstrated that the thoracic and lumbar region differ significantly and that the ranges do not overlap between the respective regions. It is also clear that TLTV has a range (95° < TLTV < 200°) that corresponds closely to the lower bound of L1 (90°) and the higher bound of T12 (210°), demonstrating the overlap of developing somites between the thoracic region resulting in the blended TLTV morphology. The results, therefore, indicate that point measurements of TLTV facets will fall into the range interval that encompasses both the thoracic and lumbar regions.

Unlike the regular vertebral segments, the range shows that TLTV is a clear combination of the thoracic and lumbar regions, as the superior facet angle encompasses the “facet intervals” of both the thoracic and lumbar region. This again, demonstrates the overlap of the developing somite. As demonstrated in the results, the superior facets of TLTV are often asymmetrical with one side resembling the thoracic region and the other side the lumbar region. Alternatively, it is possible for both facets to have intermediate superior facet placement. Regardless of its location in the vertebral column, other features may include unilateral or bilateral aplasia/hypoplasia of the transverse processes.

The results also demonstrate that unlike regular vertebral segments, TLTV are asymmetrical as demonstrated by the differences in the sizes of the superior facet angles on the left and right sides.

## 5 CONCLUSION

In conclusion, a TLTV results from overlapping developing fields and resembles both the thoracic and lumbar region. The result from this study show that TLTV can be identified by measuring the angles of the superior articular facets and comparing it to the basline of the region it is located in. The main concept being that thoracic vertebrae have a very unique morphology and confidence interval as do lumbar vertebrae. However, the results clearly show that TLTV will not fall within the bell curve of a typical vertebral segment. This anomaly has a relatively high prevalence (30%). The results strongly infer that measurements of the superior articular facet angle, also called the superior zygapophyseal facet angle, of vertebrae can differentiate between thoracic, lumbar, and transitional vertebrae skeletal remains. This will help with the correct sequencing of the vertebral column during evaluation and also help to make note of any border shifts in the vertebral column or numeric variation. This is critical to accurately note the inventory of skeletal remains during any osteological evaluation of decedent remains.

## Data Availability

Data is available on the University of Pretoria public database.
